# Genome and Transcriptome Analysis of the *Torreya grandis* WRKY Gene Family during Seed Development

**DOI:** 10.3390/genes15030267

**Published:** 2024-02-21

**Authors:** Ruiqian Zhu, Ning Gao, Jiali Luo, Wenhui Shi

**Affiliations:** 1 State Key Laboratory of Subtropical Silviculture, Zhejiang A&F University, Hangzhou 311300, China; zrq1343654864@163.com (R.Z.); gaoning9705@163.com (N.G.); ljl13588059380@163.com (J.L.); 2 Key Laboratory of Bamboo Science and Technology, Zhejiang A&F University, Ministry of Education, Hangzhou 311300, China

**Keywords:** *Torreya grandis*, WRKY family, seed development

## Abstract

*Torreya grandis*, an economically significant evergreen tree species exclusive to subtropical China, is highly valued for its seeds. However, the seed development process of *T. grandis* remains relatively unexplored. Given the pivotal role WRKY transcription factors (TFs) play in coordinating diverse cellular and biological activities, as well as crucial signaling pathways essential for plant growth and development, and the lack of comprehensive investigation into their specific functions in *T. grandis*, our study investigated its genome and successfully isolated 78 *WRKY* genes and categorized them into three distinct clades. A conserved motif analysis unveiled the presence of the characteristic WRKY domain in each identified TgWRKY protein. The examination of gene structures revealed variable numbers of introns (ranging from zero to eight) and exons (ranging from one to nine) among *TgWRKY* genes. A chromosomal distribution analysis demonstrated the presence of TgWRKY across eight chromosomes in *T. grandis*. Tissue-specific expression profiling unveiled distinctive patterns of these 78 *TgWRKY* genes across various tissues. Remarkably, a co-expression analysis integrating RNA-seq data and morphological assessments pinpointed the pronounced expression of *TgWRKY25* during the developmental stages of *T. grandis* seeds. Moreover, a KEGG enrichment analysis, focusing on genes correlated with *TgWRKY25* expression, suggested its potential involvement in processes such as protein processing in the endoplasmic reticulum, starch, and sucrose metabolism, thereby modulating seed development in *T. grandis*. These findings not only underscore the pivotal role of *WRKY* genes in *T. grandis* seed development but also pave the way for innovative breeding strategies.

## 1. Introduction

Transcription factors (TFs), renowned for their ability to selectively bind to specific sequences in the upstream promoter regions of genes, play a crucial role in regulating gene expression involved in numerous cellular processes. This regulatory function is significant not only in plants but also across all living organisms [1,2]. Within this context, the WRKY family of transcription factors is particularly noteworthy for its critical role in the complex network of plant development under diverse environmental conditions.

Initially discovered in *Ipomoea batatas* [3], WRKY proteins are now recognized as crucial regulators of plant growth, development, and response to environmental stresses. These TFs are characterized by a distinctive WRKYGQK sequence at their N-terminus, the hallmark of the WRKY domain. This domain typically comprises around 60 amino acids, featuring a four-stranded β-sheet and a zinc-finger motif, which enables specific interactions with DNA sequences known as W-box elements (5’-[T]TGAC[C/T]-3’) [4]. Although the structural elements of WRKY TFs are highly conserved, the family exhibits considerable diversity in the plant kingdom. Categorized into three primary groups—Group I, Group II, and Group III—differentiated by the number of WRKY domains and the zinc-finger motif structures [5], each group and subgroup of WRKY TFs may have unique functions and roles in plant biology, playing significant parts in responses to biotic and abiotic stresses and developmental processes. Group I proteins contain two WRKY domains, while those in Groups II and III have one. The distinguishing factor between Group II and III proteins is their zinc-finger motifs; Group II resembles Group I with the motif C-X4-5-C-X22-23-H-X1-H, whereas Group III has a C-X7-C-X23-HX1-C motif [6]. Unique to plants, WRKY genes have been identified in various species, including *Arabidopsis thaliana* with 74 WRKY proteins, *Glycine max*, and *Oryza sativa*, which has 109 WRKY proteins [7,8,9].

Recent advances in molecular biology and genomics have led to the identification of an increasing number of WRKY genes, uncovering their varied functional roles. These genes are expressed in a range of plant tissues, including roots, stems, leaves, flowers, and fruits, and are integral in regulating diverse growth and development stages. Notably, specific WRKY genes like WRKY13, WRKY33, and WRKY71 are essential in processes such as root development, stem node differentiation, leaf expansion, and fruit maturation, forming a critical molecular regulatory base for plant growth [10,11,12,13,14,15]. Additionally, WRKY genes are central to plant responses to a variety of stresses [16]. Studies have shown their inducible response to both abiotic stresses (such as drought, high temperature, and salinity) and biotic stresses (including pathogen infections and insect infestations). By modulating stress-responsive gene cascades, WRKY genes enhance plant resilience and adaptability [17]. For instance, in wheat (*Triticum aestivum*), 47 *WRKY* genes are upregulated under salt stress, and in rice (*O. sativa*) [18,19], 17 *WRKY* genes respond to drought conditions [20]. WRKY genes play a crucial role in the complex interplay of plant growth, development, and stress responses by regulating downstream target genes through DNA binding [21]. They also interact with other transcription factors and hormone signaling pathways, creating intricate regulatory networks [22]. A deeper understanding of WRKY genes’ regulatory mechanisms and associated signaling pathways promises to offer significant theoretical and practical contributions to plant biology.

*T. grandis*, a notable tree species indigenous to the subtropical mountains of China and belonging to the *Torreya* genus of the Taxaceae family, is distinguished by its seeds’ high fatty acid content, including linoleic acid, oleic acid, and other unsaturated fatty acids. These bioactive compounds are known for their cholesterol-lowering effects in human blood, offering potential benefits in preventing or mitigating cardiovascular and cerebrovascular diseases such as atherosclerosis and hypertension [23]. This highlights the species’ significant economic and health-related value. However, previous research on *T. grandis* seeds has largely concentrated on their composition and health benefits [24,25,26], while the process of seed development and the underlying regulatory mechanisms, crucial for seed yield, have not yet been fully understood.

In this study, we aimed to elucidate the regulatory role of the WRKY gene family in *T. grandis* seed development by undertaking a comprehensive identification and analysis of *WRKY* genes in the *T. grandis* genome. Our analysis included examining their chromosomal distribution, phylogenetic relationships, and conserved domains. Furthermore, a transcriptomic analysis provided insights into the expression patterns of the *WRKY* gene family across different seed development stages. This research advances our understanding of the *WRKY* gene family’s function in *T. grandis* seed development and contributes to the broader knowledge of WRKY functions.

## 2. Material and Methods

### 2.1. Plant Materials

The study utilized seeds of *T. grandis* obtained from the Botanical Garden of Zhejiang Agriculture and Forestry University in Linan City, Zhejiang Province, China. Collection of the seeds occurred monthly from May to September. Subsequently, the seeds were carefully de-scaled using a manual peeling method based on established protocols for *T. grandis* seed kernels [26,27,28], ensuring clean and intact seeds, then rapidly frozen in liquid nitrogen and preserved at −80 °C for subsequent analyses.

### 2.2. Identification of WRKY Genes in T. grandis

Genomic sequences of *T. grandis*, encompassing both nucleotide and amino acid data, were retrieved from the Figshare database [26]. An HMM search, utilizing Pfam ID PF03106, was conducted to identify the *WRKY*-DNA-binding domain, employing an E-value threshold of ≤e^−10^. This process initially identified 90 putative *WRKY* sequences in *T. grandis*. A comparative analysis was subsequently performed by juxtaposing these sequences against 72 *WRKY* gene sequences from *Arabidopsis* sourced from Phytozome V13 [23], using the blastp tool. We applied a strict e-value cutoff of ≤e^−5^ to pinpoint potential *WRKY* genes. Finally, utilizing NCBI-CDD results, we definitively identified 78 *WRKY* genes in *T. grandis*, confirming the presence of the WRKY domain in each.

### 2.3. Phylogenetic Analysis of TgWRKY and AtWRKY Gene Families

The full-length WRKY amino acid sequences from both *T. grandis* and *A. thaliana* were aligned using MAFFT version 7.520 with the default settings [29]. Maximum-likelihood phylogenetic trees were constructed with IQ-TREE v2.2.0 [30], employing the model automatically selected by the software (IQ-TREE v2.2.0, ‘Auto’ option) and 5000 ultrafast bootstraps for robustness [31]. The resulting evolutionary trees were modified and visualized using Evolview v2 [32].

### 2.4. Gene Structure Analysis of TgWRKY Gene Family

For the *WRKY* gene family in *T. grandis*, MEME Suite version 5.5.4 was employed to analyze the conserved motif structures and assess the functional significance. Functional annotations of these motifs were verified against the NCBI-CDD database [33,34]. The gene feature format file (gff3) from the *T. grandis* genome facilitated the verification of the chromosomal positions of the *TgWRKY* genes. The visualization of the findings was achieved using TBtools-Ⅱ version 2.016 [35].

### 2.5. RNA Extraction and Transcriptome Analysis

Total RNA extraction from *T. grandis* seeds was performed using the RNAprep Pure Plant Kit (DP441, Tiangen, Beijing, China). Approximately 100 mg of each sample type was immediately frozen in liquid nitrogen and subsequently pulverized into a fine dust using a mortar and pestle. Subsequently, 500 μL of pre-β-ME-added Buffer SL was introduced to the powder and vigorously vortexed. After centrifugation, the supernatant was moved to a fresh tube, followed by the addition of ethanol and another centrifugation step to remove the supernatant. The spin column was then treated with a Buffer RW1 and DNase I solution and incubated at an ambient temperature for 15 min. Following several centrifugation steps and buffer exchanges, the membrane of the spin column was dried, and the RNA was finally eluted using RNase-free water, then preserved at −70 °C for future analysis. The purity, concentration, and integrity of the extracted RNA were assessed using a NanoDrop 2000 spectrophotometer.

mRNA libraries were subsequently prepared for each sample and sequenced on an Illumina Novaseq platform. The quality assessment of the raw data was performed by discarding reads with adapter sequences, poly-N segments, and those of inferior quality. Subsequently, FastQC was employed to determine the Q20 and Q30 scores, and the high-quality reads were aligned to the *T. grandis* reference genome using HISAT2 version 2.2.1 [26,36]. The mapped reads for each gene were quantified using feature-counts software (version 1.5.0), and gene expression levels were quantified using fragments per kilobase million (FPKM) as the unit.

### 2.6. Weighted Gene Coexpression Network Analysis (WGCNA)

The R-package WGCNA was utilized to identify gene co-expression modules among the 24,548 genes obtained after FPKM normalization and filtering [37]. A soft-thresholding power (β = 10) was applied to the gene expression matrix to facilitate this analysis. The resulting modules consisted of clusters of highly interconnected genes, characterized by substantial correlation coefficients among them. To ensure the robustness of our findings, the minimum module size was set to 30, and modules with eigengenes exhibiting high correlations (a threshold of 0.25) were merged.

## 3. Results

### 3.1. Identification and Phylogenetic Analysis of WRKY Proteins in T. grandis

To elucidate the WRKY gene family in *T. grandis*, a genome-wide search was conducted using HMMER, employing *A. thaliana WRKY* sequences as reference queries. A conserved domain analysis substantiated that all identified WRKY genes in *T. grandis* exhibited either single or double WRKY domains at their N-termini, a defining characteristic of the WRKY gene family. This search resulted in the identification of 78 potential WRKY genes within the *T. grandis* genome (Table S1).

**Figure 1:**
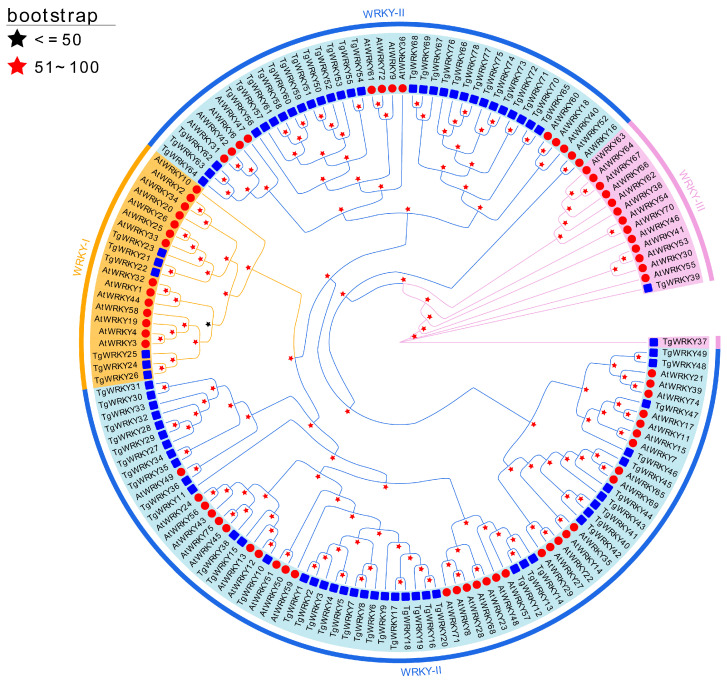
Phylogenetic relationships among WRKY proteins in *T. grandis* and *A. thaliana*. Phylogenies were deduced using a maximum-likelihood inference via IQ-TREE v2.2.0, applying the model automatically selected by IQ-TREE (‘Auto’ option) and 5000 ultrafast bootstraps.

To analyze the evolutionary relationships of TgWRKY and AtWRKY, a phylogenetic analysis was conducted using IQ-TREE. The amino acid sequences of TgWRKY from *T. grandis* were aligned with those of AtWRKY from *A. thaliana*. The analysis revealed that the 78 predicted TgWRKY proteins could be classified into three primary groups: WRKY-I, WRKY-II, and WRKY-III. Specifically, Group I contained 6, Group II comprised 70, and Group III encompassed 2 TgWRKY proteins (Figure 1).

### 3.2. Gene Structure and Synteny Analysis of TgWRKY Gene Family

**Figure 2:**
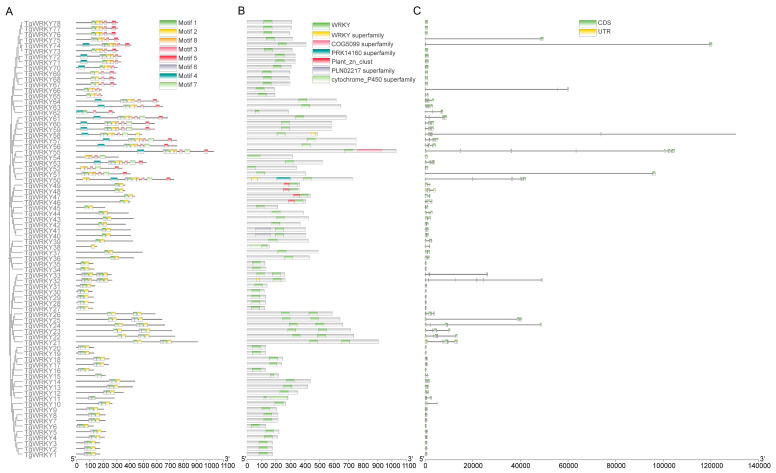
Phylogenetic relationship and gene structure of TgWRKY. (**A**) Phylogenetic relationship and motifs of TgWRKY. (**B**) The conserved structural domains of TgWRKY. (**C**) The distribution of coding sequences (CDS) in *TgWRKY*, with green boxes indicating CDS and yellow boxes denoting untranslated regions (UTRs).

The gene structure diversity of the TgWRKY gene family was investigated using annotation files from the *T. grandis* reference genome, with visualizations generated via TBtools-Ⅱ. A MEME (version 5.5.4) analysis of the conserved motifs within TgWRKY proteins identified seven distinct motifs across the 78 TgWRKY proteins. These motifs varied in number within individual proteins, ranging from one in TgWRKY38 to eight in proteins such as TgWRKY53 and TgWRKY55 (Figure 2A). It was observed that all TgWRKY proteins possessed the WRKY domain (Figure 2B).

The analysis of exon–intron structural diversity, a crucial aspect of gene family evolution, also supported phylogenetic classifications. This study’s intron and exon structure analysis aimed to enhance understanding of the phylogenetic relationships and classifications within the WRKY family. The findings indicated a variation in the number of introns (ranging from zero to eight) and exons (ranging from one to nine) across the gene family (Figure 2C).

**Figure 3:**
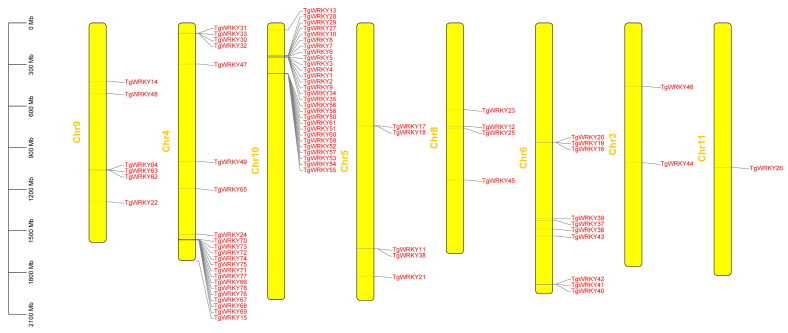
Chromosomal distribution of *TgWRKY* in *T. grandis*.

As depicted in Figure 3, the 78 identified *TgWRKY* genes were distributed across eight chromosomes of *T. grandis*. Notably, chromosomes 1, 2, and 7 lacked *TgWRKY* genes. Chromosomes 10 and 4 had the highest numbers of *TgWRKY* genes, with 28 and 22 genes, respectively. In contrast, chromosomes 11 and 3 had only one or two *TgWRKY* genes. The distribution of the remaining *TgWRKY* genes spanned the other four chromosomes, with chromosomes 6, 9, 5, and 8 hosting 10, 6, 5, and 4 *TgWRKY* genes, respectively.

### 3.3. Expression Pattern Analysis of TgWRKY in Various Tissues of T. grandis

**Figure 4:**
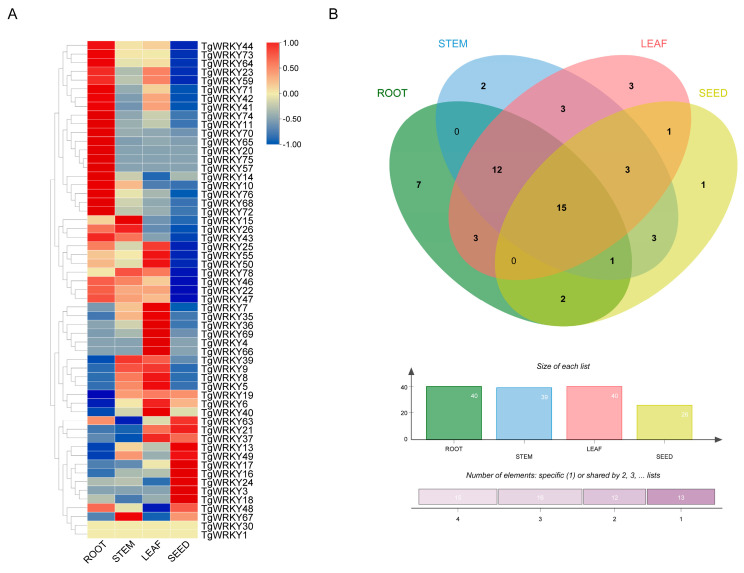
Expression patterns of *TgWRKY* in various tissues of *T. grandis*. (**A**) Heatmap displaying expression levels of *TgWRKY* in different tissues. (**B**) Venn diagram showing the number of *TgWRKY* expressed in various tissues.

To elucidate the functions of *TgWRKY* genes in the seed development of *T. grandis*, we analyzed the expression profiles of these genes across different organs, including stems, leaves, roots, and seeds, using transcriptomic data (Table S2). Our findings revealed that 51.3% (40 out of 78), 50.0% (39 out of 78), 51.3% (40 out of 78), and 33.3% (26 out of 78) of the *TgWRKY* genes were expressed in roots, stems, leaves, and seeds, respectively (Figure 4B). Notably, 15 *TgWRKY* genes exhibited expression in all these tissues, while the expression patterns of the other *TgWRKY* genes significantly varied across stem, leaf, root, and seed tissues. In stems, *TgWRKY67* showed the highest expression levels, while *TgWRKY63* was characterized by the lowest expression (Figure 4A). In leaves, *TgWRKY66* was highly expressed, as opposed to *TgWRKY48*, which showed low expression. *TgWRKY68* displayed high expression in roots, while *TgWRKY19* had a lower expression level than the average. Additionally, in seed tissue, *TgWRKY18* exhibited the highest expression, contrasting with *TgWRKY47*, which showed the lowest expression.

### 3.4. Expression Pattern Analysis of TgWRKY during the Seed Development of T. grandis

**Figure 5:**
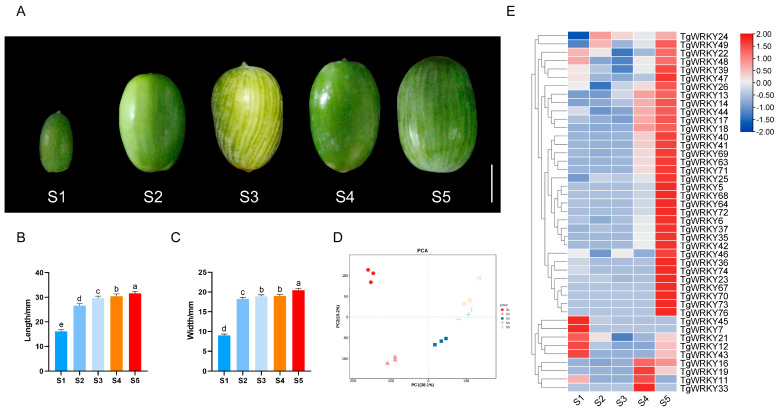
Transcriptomic and morphological characteristics of *T. grandis* seeds at different developmental stages. (**A**) Seed photographs. The scale bar is 1 cm. Seed length (**B**) and width (**C**) measurements, with error bars representing the standard deviation (SD) for 15 samples from three trees. Different letters denote significant differences (*p* < 0.05) as determined by a one-way ANOVA with Tukey’s post hoc test. (**D**) A principal component analysis (PCA) of the gene expression dataset in ovulate strobilus and (**E**) a heatmap of *TgWRKY* expression during seed development.

To elucidate the role of TgWRKY genes in the seed development of *T. grandis*, seeds were collected at five developmental stages, designated S1–S5, from May to September. A morphological analysis identified distinct patterns of development: a phase of rapid expansion from S1 to S2, followed by a relatively stable period from S3 to S5 (Figure 5A). Supporting these morphological findings, statistical analyses of seed length and width further illustrated the developmental progression of *T. grandis* seeds (Figure 5B,C). These stages formed the basis for the subsequent RNA-seq analysis.

A principal component analysis (PCA) confirmed a strong correlation among the three replicates for each sample, affirming data reliability (Figure 5D). Notably, PCA segregated the samples into three distinct clusters: S1, S2, and S3–S5, aligning with the developmental stages. As illustrated in Figure 5E and Table S3, 55.1% (43 out of 78) of *TgWRKY* genes were expressed across the various developmental stages. Among these, 20 *TgWRKY* genes, such as *TgWRKY5*, *TgWRKY13*, *TgWRKY14*, *TgWRKY18*, and *TgWRKY25*, exhibited a marked increase in expression. Conversely, only two *TgWRKY* genes, *TgWRKY45* and *TgWRKY7*, showed limited expression during these stages, suggesting their potential roles in seed development in *T. grandis*.

### 3.5. Construction of the Regulatory Network Associated with Seed Development in T. grandis

**Figure 6:**
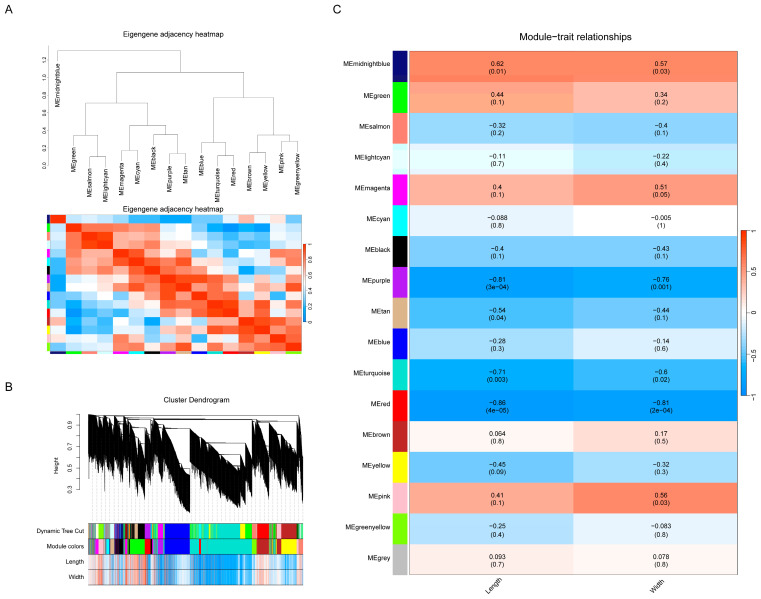
Construction and analysis of a weighted gene co-expression network. (**A**) Eigengene adjacency heatmap. (**B**) Cluster dendrogram. (**C**) Heatmap showing module-trait correlations.

A weighted gene co-expression network analysis (WGCNA) is a systems-biology approach that focuses on networks of strongly associated genes rather than individual genes. This method has been effectively employed in various genomic studies. After filtering the data, a total of 24,548 genes with FPKM values were subjected to analysis using the R-package WGCNA. This analysis revealed fifteen distinct modules (Figure 6A), visualized as tree branches in a hierarchical clustering dendrogram, with each leaf representing a gene (Figure 6B). The modules were displayed in various colors (Figure 6B). A correlation analysis, illustrated in Figure 6C, was conducted between seed length, width, and the fifteen modules. The chart’s color shading (red for positive and blue for negative correlations) indicated significant correlations between these modules and the seed dimensions. A module-trait association analysis identified the ‘red’ module, comprising 1203 genes, as potentially involved in seed size increases due to its strong correlation with length and width. Notably, *TgWRKY25* was part of the ‘red’ module, suggesting its potential role in *T. grandis* seed development.

**Figure 7:**
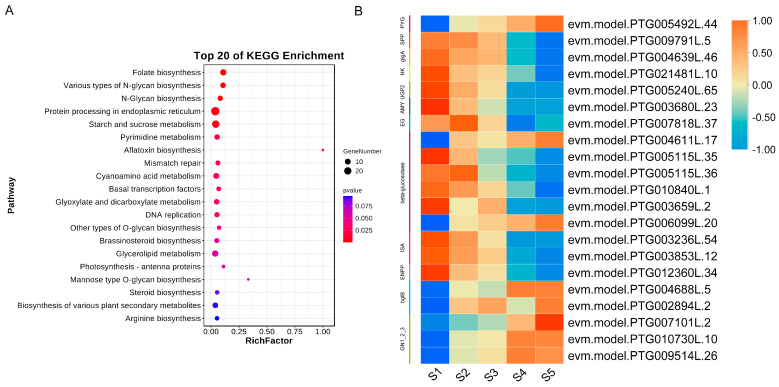
DEGs related to *TgWRKY25* during the seed development. (**A**) KEGG analysis of DEGs related to *TgWRKY25* expression. (**B**) Heatmap of DEGs related to starch and sucrose metabolism.

Furthermore, to explore the potential regulatory network of *TgWRKY25* in seed development, we conducted a KEGG enrichment analysis on the genes within the ‘red’ module where *TgWRKY25* is located. The KEGG enrichment analysis of these genes, as depicted in Figure 7A, highlighted enriched pathways including those for protein processing in the endoplasmic reticulum, starch and sucrose metabolism, pyrimidine metabolism, among others. Among these pathways, 21 of 1203 genes involved in starch and sucrose metabolism, such as *TgPYG* (evm.model.PTG005492L.44) and *Tgbata-glucosidase* (evm.model.PTG0066099L.20), exhibited a significant increase during seed development (Figure 7B and Table S4). This suggested that these genes might play essential roles in the seed development of *T. grandis*.

## 4. Discussion

*T. grandis* seeds offer significant dietary benefits, with their yield and nutritional value varying depending on their developmental stage [38]. The molecular regulation of seed development involves a complex interplay of various factors and signaling pathways, encompassing plant hormone regulation (such as gibberellins, auxins, abscisic acid, ethylene, etc.), transcription factor control (such as LEC1, LEC2, FUSCA3, etc.), nutrient transport and storage, cell division and expansion, and signaling transduction pathways [39,40,41,42,43]. These regulatory factors and pathways interact with each other to collectively regulate the process of seed development, ensuring the normal development of seeds in morphology, structure, and function. There exists a notable correlation between these molecular regulatory pathways and the WRKY gene family. Previous research has highlighted the involvement of WRKY transcription factors in crucial processes of seed development, such as seed morphogenesis, endosperm development, and the differentiation of endosperm and seed coat tissues [44]. WRKY TFs achieve this by modulating plant hormone synthesis, signal transduction pathways, and responsive gene expression. For instance, WRKY TFs interact with various plant hormone signaling pathways, including gibberellins, auxins, abscisic acid, and ethylene, to regulate downstream gene expression, thereby influencing processes such as nutrient accumulation in seeds, endosperm development, and seed coat rupture [45]. Moreover, WRKY TFs regulate genes associated with cell cycle regulation, impacting the rate, direction, and pattern of cell division and expansion, ultimately affecting seed size, quality, and morphology [46]. Therefore, the WRKY family plays a pivotal role in the molecular regulatory pathways of seed development.

The number of identified *WRKY* genes varies among different plant species, such as *A. thaliana*, *O. sativa*, *Arachis hypogaea*, *Cucumis sativus*, *Liriodendron chinense*, *Eucommia ulmoides*, and others [11,12,16,47,48,49,50,51]. Despite their ubiquitous presence across different plants, the quantity and classification of WRKY families vary among species, reflecting the diversity of plant genomes. For example, *A. thaliana* has approximately 74 WRKY members, instrumental in key plant processes like growth, development, and environmental stress responses [52], whereas rice (*O. sativa*) has over 100 known WRKY members [53]. These variations may result from environmental pressures and evolutionary forces, leading to distinct evolutionary trajectories within the gene family [54]. These differences suggest gene amplifications or reductions in specific lineages [5]. In our study, 78 TgWRKY family members were identified in *T. grandis*, categorized into three groups, WRKY-I, WRKY-II, and WRKY-III (Figure 1), and distributed across eight chromosomes (Figure 3). Although similar to that in *Arabidopsis*, the number of *WRKY* genes in *T. grandis* is fewer than in rice, suggesting potential gene amplifications or reductions in specific lineages. Furthermore, our findings revealed closely related orthologous *WRKY* genes between *T. grandis* and *A. thaliana* (e.g., *TgWRKY10* and *AtWRKY112*; *TgWRKY36* and *AtWRKY49*; *TgWRKY38* and *AtWRKY45*/*AtWRKY75*; *TgWRKY45* and *AtWRKY65*/*AtWRKY69*; *TgWRKY47* and *AtWRKY11*/*AtWRKY17*), indicating a common set of ancestral *WRKY* genes before their divergence, highlighting a close relationship between *T. grandis* WRKY proteins and those of *Arabidopsis*. Despite this, the variations in *WRKY* gene quantity and classification among different species emphasize the diversity and adaptability of plant genomes.

WRKY proteins, prevalent in plants, play pivotal roles in vital biological processes, including growth, development, and stress responses [52,55]. In *Taxus*, another genus within the Taxaceae family, 61 WRKY transcripts were identified from *Taxus chinensis* transcriptome datasets, with certain WRKY genes significantly enhancing the expression levels of taxol-biosynthesis-related genes [56]. In our study, 26 WRKY members were detected in *T. grandis* seeds, with the expression levels of 20 *TgWRKY* genes (including *TgWRKY5*, *TgWRKY13*, *TgWRKY14*, *TgWRKY18*, *TgWRKY25*, etc.) varying during seed development (Figure 5). It is notable that the number of WRKY family members is relatively small in the *T. grandis* seed. This may be attributed to the fact that different plant tissues may possess distinct biological functions and metabolic demands, thus resulting in varying requirements for WRKY family members [57]. Certain tissues may necessitate a greater number of WRKY genes to regulate specific biological processes, while others may require fewer. Additionally, gene expression levels are regulated by genetic and environmental factors, which may lead to differential expression levels of *WRKY* genes across different tissues [58]. Nevertheless, a KEGG enrichment analysis indicated the WRKY family’s significant role in regulating key biological processes, underscoring the important impact of WRKY genes on seed growth and development.

Additionally, through a co-expression analysis and morphological assessments, it was found that *TgWRKY25* was closely related to the seed development of *T. grandis* (Figure 6). These results strongly suggested the crucial role of *WRKY* genes in regulating seed development in *T. grandis*. WRKY transcription factors (TFs) regulate gene expression by binding to the DNA of target genes via the WRKY domain [59]. For example, in *Arabidopsis*, *AtWRKY12* and *AtWRKY13* regulate the expression of the downstream gene *AtFUL*, thus influencing flowering [60]. Here, our study found that 21 genes belonging to the ‘red’ module, such as *TgPYG* (evm.model.PTG005492L.44), *TgEG* (evm.model.PTG007818L.37), *TgGN1_2_3* (evm.model.PTG007101L.2), etc., which were involved in starch and sucrose metabolism (two crucial carbon sources playing pivotal roles in seed maturation), correlated with *TgWRKY25* expression. These suggested the potential role of TgWRKY25 in regulating genes related to starch and sucrose biosynthesis/metabolism, thereby affecting seed development in *T. grandis*. Future research should elucidate the function and regulatory mechanism of WRKY in the seed development of *T. grandis*. 

## 5. Conclusions

In this study, we conducted a comprehensive analysis of the WRKY gene family in the *T. grandis* genome, resulting in the identification of 78 *TgWRKY* genes distributed across eight chromosomes and classified into three distinct clades. Through a conserved motif analysis, we confirmed the presence of the characteristic WRKY domain in each *TgWRKY* gene. Additionally, a gene structure analysis revealed variability in the number of introns and exons among *TgWRKY* genes, which ranged from zero to eight and one to nine, respectively, highlighting structural diversity within the gene family. A chromosomal distribution analysis indicated the dispersed localization of *TgWRKYs* across *T. grandis* chromosomes. Moreover, our investigation into tissue-specific expression patterns unveiled differential expressions of these 78 *TgWRKYs* across various tissues, providing insights into their potential roles in different physiological processes. Furthermore, a co-expression analysis, integrating RNA-seq data with morphological assessments, identified TgWRKY25 as closely associated with seed development in *T. grandis*. These findings underscored the crucial role of WRKY genes in the seed development of *T. grandis*, shedding light on their regulatory mechanisms and functional significance. Furthermore, our study paves the way for future research avenues, offering new prospects for the breeding and genetic improvement of *T. grandis*.

## Data Availability

The original contributions presented in the study are included in the article/supplementary material, further inquiries can be directed to the corresponding author.

## Supplementary Materials

The following supporting information can be downloaded at: https://www.mdpi.com/article/10.3390/genes15030267/s1, Table S1. The ID numbers and names of WRKY genes in *T. grandis* and *A. thaliania*. Table S2. The FPKM values of *TgWRKY* genes in various tissues of *T. grandis*. Table S3. The FPKM values of *TgWRKY* genes in various seed development stages in *T. grandis*. Table S4. DEGs related to sucrose and starch metabolism.
